# Retrospective Cohort Study Comparing Outcomes and High-Risk Factors of Patients Presenting with Necrotizing Soft Tissue Infections in Far North Queensland—20 Years of Experience

**DOI:** 10.3390/tropicalmed10100300

**Published:** 2025-10-21

**Authors:** Sarah Whitehouse, Ju Yong Cheong, Heng-Chin Chiam

**Affiliations:** 1 Cairns and Hinterland Hospital and Health Service, Cairns, QLD 4870, Australia; juyong.cheong2@health.qld.gov.au (J.Y.C.); heng-chin.chiam@health.qld.gov.au (H.-C.C.); 2 Cairns Clinical School, School of Medicine, James Cook University, Cairns, QLD 4814, Australia

**Keywords:** soft tissue infection, epidemiology, surgery, necrotizing fasciitis, Australia

## Abstract

Background: Necrotizing soft tissue infections (NSTIs) are high-morbidity and high-mortality conditions that particularly affect comorbid patients. Previous Australian cohorts had limited numbers of patients within them; however, due to geographical, social and climate factors, North Queensland has significantly higher presentations than most of the country. Methods: We have completed a retrospective cohort study between 2002 and 2012 and 2014 and 2024 of patients with ICD codes and documented clinical histories consistent with NSTI who presented to a single tertiary centre. Results: 213 patients were identified. There was a 14% mortality rate, and common comorbidities were diabetes, smoking, high BMI and high ethanol use. Patients were likely to present with vital signs within the normal range and high inflammatory markers. Of the patients, 51% identified as First Nations, an incidence rate 88 times higher than all other ethnicities put together. First Nations patients were younger (51.78 vs. 55.74 years, *p* = 0.02), had higher rates of diabetes (86% vs. 34%, *p* ≤ 0.001), shorter times spent in ICU (6.77 days vs. 10.1 days, *p* = 0.03), higher average time to theatre (57.7 h vs. 35.3 h, *p* = 0.03) but a comparable mortality rate (13.9% vs. 13.3%, *p* ≥ 0.99) Conclusions: This study helps us to better understand NSTI in the Australian setting as a basis for further research.

## 1. Introduction

Necrotizing soft tissue infections (NSTIs) are high-morbidity and high-mortality conditions which require rapid diagnosis and aggressive treatment to improve outcomes. Usually polymicrobial, they rapidly spread through subcutaneous tissue, fascia or muscle, causing sepsis. Nomenclature can change for various parts of the body; for example, infections of the perineum are referred to as Fournier’s gangrene. However, diagnostic and treatment principles remain the same regardless of site or depth of infection [1]. Susceptible patients are often comorbid, with the most common association being diabetes mellitus [2,3,4]. Peripheral neuropathy and medication side effects in these patients can lead to difficulty in detecting the classical symptoms of NSTIs [5].

Surgical exploration, or ‘cut down’ or ‘finger test’, is considered the gold standard for the diagnosis of NSTIs where macroscopic dishwater fluid, necrotic tissue, lack of bleeding and a positive finger sweep test are identified [1,4,6,7,8].

Current guidelines outline the mainstay of treatment as early debridement of all infected tissue until only healthy tissue is exposed [1,2,6,8,9,10,11,12]. Empirical antibiotics, resuscitation and supportive care are instigated simultaneously with aggressive debridement [6,8,11]. Delay in diagnosis and delay to surgery have been seen to increase mortality [3]. Reconstruction only occurs after confirmation that there is no further necrotic tissue present. Adjuncts to this treatment course such as intravenous immunoglobulin and hyperbaric oxygen chambers have been controversial [8,13].

Previous studies in Australian cohorts have had limited numbers of patients. Anecdotally, due to geographical, social and climate factors, North Queensland has higher presentations than the rest of the country.

The Cairns Hospital is the surgical referral centre for a significant portion of Far North Queensland. The geographical catchment covers Tully in the South, Cape York and the Torres Strait in the North and Cairns and the Cairns Hinterland in the East and West, covering most of the Atherton Tablelands. This is a mix of tropical coastal environments usually with very high humidities, tropical wet and dry seasons, dry desert environments and isolated tropical island settings. Distances between primary presentation clinics and Cairns Hospital can be up to 850 km and require a mix of boat, rotor and fixed-wing transport to transport patients to a surgical service that can provide definitive treatment.

Health equity of Australia’s First Nations people is a vital topic for surgeons. Australian First Nations people have poorer health outcomes over an almost universal front compared to the general Australian population. There is significant funding through initiatives such as ‘Closing the Gap’ to help rectify this discrepancy. The Torres and Cape Hospital and Health Service, covered by Cairns Hospital, has the highest percentage of First Nations patients in Australia (over 70%), with over 16,000 individuals and 60 different traditional owner groups [14]. This number is approximately 12% of self-identifying First Nations patients for the greater Cairns and Hinterland Health Service, higher than the Australian average [15].

The objectives of this study were to explore the epidemiology of this cohort further, in order to determine the presence of any specific comorbidities, presentation patterns with observations and blood work or outcomes which further intervention could be targeted to.

## 2. Materials and Methods

A retrospective observational cohort study was completed to analyze all patients presenting with NSTI was compiled between 2014 and 2023 who were treated at a single tertiary institution in Far North Queensland, Cairns Hospital. Patients were identified by searching for compatible ICD codes for NSTIs. All paper and electronic charts were reviewed individually by the authors and included if documented clinical findings were documented as consistent with NSTI (i.e., dishwater fluid, infection running along fascial planes or described as NSTI in operative findings). Patients were excluded if their presentation was not in keeping with NSTI findings; these included presentations for dialysis care post-acute presentation, pancreatitis presentations, and severe abscess or cellulitis presentations. The appropriate demographic, presenting observation, blood work and outcome data points were extracted from each patient who met these criteria. This time point was identified due to a change in data storage systems between 2013 and 2014. These data points were added to a previous study completed at the same institution, collected between 2002 and 2012.

Univariate and multivariate analyses were undertaken to find relevant links between data points, utilising Microsoft Excel and freely available statistical calculators. Subgroup analysis was performed comparing mortality and non-mortality patients, and First Nations patients with other ethnic groups. Subgroup analysis used the previously obtained data points as a comparison between the two groups. Data best represented with averages was compared with Student’s t-test. Binary categorical data were compared using the Fisher test. Categorical data with more than two categories were compared with the chi-square test of independence. Each analysis used a *p* value of 0.05 as a requirement for statistical significance.

## 3. Results

### 3.1. Patient Presentations over Time

**Figure 1:**
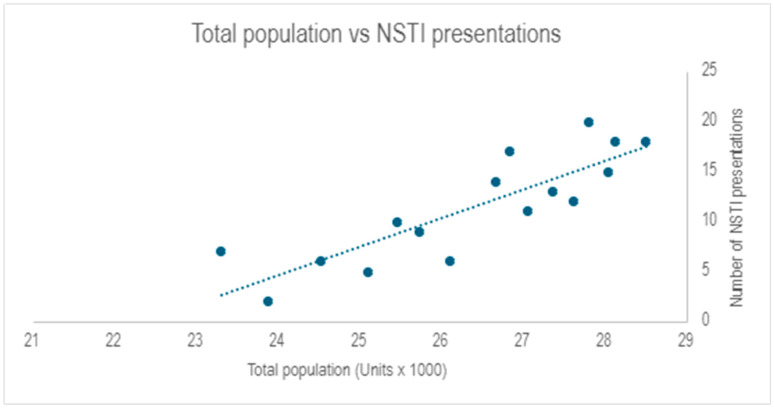
Total area population vs. NSTI presentations. The scatter plot shows the relationship between the total population of the Cairns Hospital Catchment areas (in units of ×1000 people) compared to the number of NSTI presentations occurring.

A total of 213 patients were identified across two separate data collection periods and compared to population data for the same time period, which was obtained from the Far North Queensland Regional Organization of Councils (FNQROC) [16]. Composite analysis with linear regression showed a significant correlation between population and presentations with NSTIs (r^2^ = 0.7292) (Figure 1). Linear regression also compared mortality numbers over time, showing that there was no correlation with mortality rate (r^2^ = 0.02). 

### 3.2. Patient Demographics and Comorbidities

Of the 213 patients, 131 patients identified as male (62%) and 82 (38%) as female. Self-reported ethnicity identified a total of 44 patients who identified as Aboriginal (21%), 54 as Torres Strait Islanders (25%) and 10 as both Aboriginal and Torres Strait Islanders (5%). These groups are collectively referred to as First Nations Australians. Of the patients, 29 (14%) succumbed to NSTIs or other complications before discharge. The minimum age of patients presenting was 11 years old, and the maximum age was 90. The mean age of patients was 53.7 years. (Table 1)

**Figure 2:**
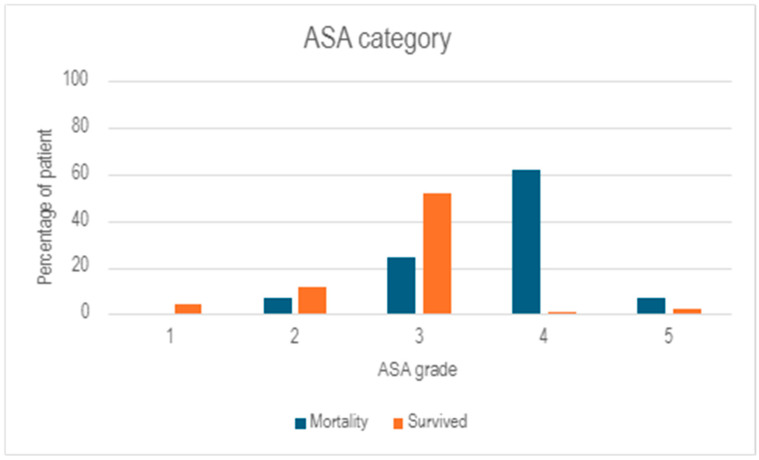
ASA category comparing the mortality and survival subgroup analysis. This graph shows the ASA category in total numbers of patients per group. The chi-square test of independence shows a *p* value of <0.0001, showing a significantly different skew of this graph.

Immunosuppression was defined as taking medications designed specifically to affect immune system functions or having conditions such as HIV that limit immunity. Sixteen patients (7.5%) fit this criterion. A patient was considered diabetic or insulin-dependent if already diagnosed or were taking treatment prior to presentation, but not if diagnosed during admission. Insulin-dependant patients are a subgroup of diabetic patients and thus count towards both demographics. Patients were considered to be smoking at any current participation level at presentation. Ethanol use was considered if patients were having any level of ethanol on a regular basis due to discrepancies on how thorough clinician documentation was in this area. ASA break down is shown in Figure 2.

### 3.3. Preoperative Work up

**Figure 3:**
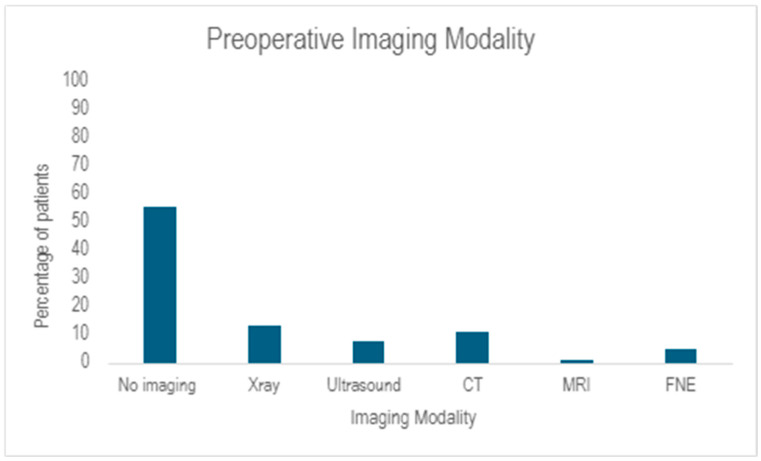
Preoperative Imaging Modality. This graph shows the type and frequency of imaging underwent by patients prior to their first operation. CT—computed tomography, MRI—magnetic resonance imaging, FNE—flexible nasoendoscopy.

All 213 patients (100%) received some form of intravenous antibiotic treatment prior to induction for the first operation. Of these, 119 patients (55.9%) proceeded to the operating theatre based purely on clinical diagnosis, rather than imaging. Of those who did have preoperative imaging, 28 (13%) had plain film x-ray, 24 (11%) underwent CT scans and 16 (7.5%) underwent ultrasound. (Figure 3)

### 3.4. Overall Operative Interventions

The number of trips to the theatre for patients ranged from 0 to 16, with the mean being 4.7 and the median being 4. (Table 2) The shortest time between presentation and surgery was 0.42 h, with the longest being 613.03 h (mean 46.68 h). The median time to theatre was 14 h with an interquartile range of 6.55–47.5 h. There were 27 outliers, all at the high end of the range.

105 patients (49%) had planned relook operations between 24 and 48 h noted in their original postoperative instructions. A total of 30 patients (14%) underwent limb amputation of either the upper or lower limb during their treatment course. Not all amputations were at index operation. 

After debridement, a range of options were chosen for reconstruction; the majority (110 patients or 52%) underwent healing with secondary intention and negative pressure dressings. 

### 3.5. Presentation Observations and Baseline Bloodwork

The first set of vital signs recorded were used for analysis, whether this was at triage in the Emergency Department, a peripheral institution or during transfer. Blood work was included with similar principles and excluded if taken after first debridement in theatre.

Of the patients, 71.8% met SIRS criteria by having at least two of the following: temperature above 38 degrees or below 36 degrees celcius, heart rate above 90, respiratory rate above 20, and/or white cell count above 12 or under 4. Average heart rate (99.76 beats per minute) and respiratory rate (20.04 breaths per minute) were on the upper end of normal accepted rates. Otherwise, there were no deviations on normal or average rates. 

Routine blood work shows significant inflammatory responses with high average white cell counts (18.43) and CRPs (292.92). Hyponatremia (Sodium 131.97) was common within this population, as was poor renal function (average urea 13.56, creatinine 216.67 and eGFR 49.65). Most patients’ acid base status was compensated (pH 7.35) despite having an average elevated lactate (2.92).

### 3.6. Two-Variable Analysis

Logarithmic regression was run between variables to determine their correlation with the Pearson’s correlation coefficient calculator.

There was no correlation between length of admission and time in ICU (r = 0.25, *p* ≤ 0.001), time to surgery (r = 0.16, *p* = 0.02) or trips to theatre (r = 0.29, *p* ≤ 0.001).

Time to theatre was not related to presentation systolic blood pressure (r = 0.094, *p* = 0.17), mean arterial pressure (r = 0.068, *p* = 0.32), heart rate (r = −0.09, *p* = 0.19), respiratory rate (r = 0.04, *p* = 0.56) or white cell count (r = −0.04, *p* = 0.56).

### 3.7. Subgroup Analysis: Mortality vs. Survival

Of the patients, 29 succumbed to NSTI during their index inpatient stay and were included in the mortality subgroup. These patients were compared to the 184 patients who survived until discharge. The average age of patients who were in the mortality group was higher (59.55 years compared to 52.82, *p* = 0.004) as well as having a reduced length of stay (30.28 days compared to 46.53 days, *p* = 0.004). They trended towards having higher admission rates to ICU (79% compared to 63%), however, this finding was not statistically significant. Overall, individual comparisons between patient comorbidities were similar but not statistically significant, except for insulin dependence which was higher in the survival group (26% compared to 7.4%, *p* = 0.03) (Table 3). However, the American Society of Anesthesiologist physical status classification system (ASA) was overall skewed towards a higher grade (*p* ≤ 0.0001) (Figure 3).

Systolic blood pressure (109.9 vs. 120.3, *p* = 0.016), diastolic blood pressure (60.24 vs. 69.21, *p* = 0.003) and mean arterial pressure (76.4 vs. 86.2, *p* = 0.003) were all lower in patients who were in the mortality group without significant differences in the other presentation vital signs. The mortality group also showed lower hemoglobin (109 compared to 124, *p* = 0.011) without significant differences in inflammatory markers or acid base parameters. Kidney function was overall lower at presentation in the mortality group (eGFR 30.9 compared to 52.5, *p* ≤ 0.0001) without significant differences in any other electrolytes. 

### 3.8. Subgroup Analysis: First Nations vs. Non-First Nations Patients

Of the patients, 108 self-identified as First Nations people and were compared to the 105 patients who identified as being of other ethnic backgrounds. This representation is approximately 5× in this group compared to the 11.5% of patients identifying as First Nations people in the FNQROC in the 2021 census. This leads to an incidence rate of 88 times higher in the First Nations population, utilizing the population data over the census periods involved in this study.

Table 4 describes how First Nations patients presented at a younger age (51.78 years compared to 55.74 years, *p* = 0.02) and were more likely to be female (45% compared to 31%, *p* = 0.05). They spent less time in the ICU (6.77 days compared to 10.1 days, *p* = 0.03) despite a similar ICU admission rate (60% compared to 69%, *p* = 1). First Nations patients also had delayed time to theatre (57.7 h compared to 35.3 h, *p* = 0.03) but had reduced numbers of trips to theatre (4.3 compared to 5.1, *p* = 0.03). First Nations patients were more likely to present with all of the measured comorbidities except immunosuppression (6.5% compared to 8.6%, *p* = 0.6); however, this did not affect the overall mortality rate (13.9% compared to 13.3%, *p* = 1). The ASA distribution curve showed no significant difference (*p* = 0.49).

## 4. Discussion

Our study has a larger cohort of patients compared to other Australian-based studies, giving us the ability to look more specifically at NSTIs in the Australian context [17,18,19]. Proud et al., as a study is of comparable size, but its inclusion criteria is the whole state of Victoria. Stewart et al. is larger found; however, it only reviewed mortality [3,20]. Our local First Nations population percentage of 11.5% is also significantly higher than the Australian average of 3.8%, allowing for a review specifically on this subgroup [21].

Overall, the general increase in cases per year mirrors Cairns’ increasing population throughout this period, as suggested by the 73% correlation. Anecdotal evidence from experienced clinicians in our institution suggest that other likely contributing factors include the significance and length of wet season per year and the exposure to natural disasters such as flooding, which are endemic to the tropics. These are difficult to account for in a retrospective study, but clinicians practicing in the local area will anecdotally quote these as environmental concerns for an increased incidence of NSTIs. Recent retrospective analysis does show increasing mortalities since 2019 locally [22]. During the analysis there was no correlation between time course and other metrics noted in the study. Of particular interest is that cases did not increase or decrease around the COVID-19 era of 2020–2021.

Overall diagnosis of NSTIs was seen as a clinical diagnosis in 55.9% of cases, rather than radiological diagnosis, in line with traditional teaching [3,7]. When radiology was used, it was an adjuvant to treatment. Other comparable studies have only shown clinical diagnosis at 36.1% [17].

The overall cohort had a higher percentage of First Nations patients than other Australian cohorts (36% or not mentioned) but is comparable to Bailey et al. [17,18,19,20,21]. This cohort of published NSTI patients has higher rates of diabetes (34.25–55.6%) and smoking (23.29–36.1%). These numbers are significantly higher than the Australian average, 5.3% diabetics and 10.6% smokers [23,24]. Australian mortality rates vary greatly (2.7–25%). Trips to theatre in other studies range from an average of 3–5.4. Amputation rates are quoted as between 10 and 23% [17,18,19,20,21]. International literature quotes amputation rates of up to 25% and mortality between 10 and 20% [8,25]. Diabetes rates as high as 70.8% are documented [2]. Cardiovascular disease and alcohol use are in keeping with Australian data [8,17,18,19,20,21,25].

The total patient cohort shows a propensity to present with SIRS, however not with the profound shock often a part of traditional teaching [8]. This shows the importance of a high level of suspicion when examining patients to prevent a missed diagnosis. 

Our study is in line with many others, showing importantly that hyponatremia may be used in conjunction with increased inflammatory markers when making this diagnosis. 

Whilst time to theatre did not cause a statistically significant difference in mortality, the significant increase in time to theatre in the mortality group may have contributed to poor outcomes, perhaps in line with traditional teaching [2,3,6,8,9,10,11]. This could be suggestive of higher percentages of patients in the mortality group being from outside the Cairns postcodes of 4870, 4878 and 4879 (72% vs. 67%). Due to the nature of life in community or on large properties, specificity with postal addresses is low outside of major settlements and therefore a poor way to determine their proximity to the hospital than more traditional techniques (e.g., kilometers between two points or travel time). Individual comorbidities did not cause a statically significant difference in mortality, while an increase in the skew of the ASA curve did, supporting McHenry et al.’s finding that mortalities were more likely to occur in patients with cumulative organ failure [9]. Low levels of insulin-dependent diabetics in the mortality group may suggest a degree of non-compliance or under-prescribing of treatment. Low intervention compliance levels are replicated regularly in studies discussing First Nations health, suggesting that this may contribute to their increased incidence. However, this is a small data set and a very difficult population, due to the remote nature of communities, to obtain sufficient accurate data and to make confident observations on.

Of the patients presenting from Cape York, the Torres Strait or from the Gulf, 82% were First Nations patients, significantly contributing to their longer time to theatre. This is an actionable area for raising awareness at primary healthcare facilities, improved communications with tertiary centres and improved transfer facilities that could decrease mortality rates further. 

Documentation does not elucidate the reasons behind the decreased trips to theatre for First Nations patients.

Despite the increased comorbidities in First Nations patients, their mortality rate was comparable to other ethnic groups. However, First Nations patients were grossly overrepresented in the overall patient cohort. This should be an area for further monitoring as ongoing work to improve the overall health of First Nations people continues. 

The strength of this study primarily lies in its size, being large compared to most Australian studies. Our long collection period allows us to note no significant changes in outcomes despite changes in medicine throughout this time period.

The retrospective nature of our study did lead to some incompleteness in collection or the inability to use standardized collection over the whole period, and required some data points not to be included in the end analysis (such as pathogen growth). Also due to changes in data storage systems over time, such as data collection costs and appropriate storage of older files, we do have a small period in the middle where we were unable to collect data from patients. Patients in our institution were also not able to receive more experimental treatments such as hyperbaric oxygen or intravenous immunoglobulin.

Future research in this area should focus on targeted implementation of disease prevention techniques in susceptible communities. 

## 5. Conclusions

Our study is a retrospective cohort study of 213 NSTI patients with data collected over 20 years, showing patients to present typically with raised inflammatory markers, hyponatraemia, poor kidney function and preserved acid base parameters. Their initial general clinical picture is not in keeping with a profoundly shocked state.

## Figures and Tables

**Table 1:** Full cohort patient demographics and comorbidities; n = 213.

Demographic/Comorbidity	Total Number	Mean or Percentage
Age		53.73 years
Gender ratio—Male	131	62%
First Nations Percentage	108	51%
Mortality	29	14%
Ischemic Heart Disease	43	20.2%
Dialysis-Dependent	18	8.5%
Immunosuppression	16	7.5%
Diabetes Mellitus	130	61%
Insulin-Dependent Diabetes	49	23%
Ethanol Use	81	38%
Smoking	86	40.4%
Body Mass Index > 30	85	39.9%

Demographics of the full cohort of patients, detailing high-risk comorbidities.

**Table 2:** Operative interventions; n = 213.

Operative Measure	Mean or Percentage	95% Confidence Interval
Number of trips to operative theatre	4.7	4.26–5.14
Time between presentation and surgery (hours)	46.68	35.46–57.89
Planned relook operation	49%	42–56%
Limb amputation	14%	10–19%

Table describing the frequency and plan of operative interventions.

**Table 3:** Comparison between mortality and survival groups.

Demographic/Comorbidity	Mortality	Survival	*p* Value
Age (years)	59.55	52.82	0.004
Gender Ratio—Male	48%	60%	0.16
Length of Admission (days)	30.28	46.53	0.004
First Nations Percentage	51%	50.5%	>0.99
ICU Admission Rate	79%	63%	0.10
Time in ICU (days)	14.83	7.26	0.018
Time to Theatre (hours)	67.85	43.44	0.08
Trips to Theatre	3.24	4.93	0.008
Ischemic Heart Disease	38.8%	19%	0.32
Dialysis-Dependent	12%	8%	0.72
Immunosuppression	14%	6.5%	0.24
Diabetes Mellitus	72%	59%	0.22
Insulin-Dependent Diabetes	7.4%	26%	**0.03**
Ethanol Use	31%	39%	0.54
Smoking	34%	41%	0.54
Body Mass Index > 30	41%	40%	>0.99

*ICU*—Intensive Care.

**Table 4:** Comparison between First Nations and Non-First Nations subgroups.

Demographic/Comorbidity	First Nations	Non-First Nations	*p* Value
Age (years)	51.78	55.74	0.02
Gender Ratio—Male	55%	68%	0.05
Length of Admission (days)	47.25	41.46	0.14
Mortality Rate	13.9%	13.3%	>0.99
ICU Admission Rate	60%	69%	0.02
Time in ICU (days)	6.77	10.1	0.03
Time to Theatre (hours)	57.7	35.3	0.03
Trips to Theatre	4.3	5.1	0.03
Ischemic Heart Disease	26%	14%	0.04
Dialysis-Dependent	13%	2.9%	0.005
Immunosuppression	6.5%	8.6%	0.6
Diabetes Mellitus	86%	34%	<0.001
Insulin-Dependent Diabetes	37%	7.6%	<0.001
Ethanol Use	34%	26%	0.25
Smoking	45%	34%	0.12
Body Mass Index > 30	41%	38%	0.38

*ICU*—Intensive Care.

## Data Availability

De-identified data is available on request to the authors, in line with the ethics approval.

## Abbreviations

The following abbreviations are used in this manuscript:

ASA	American Society of Anesthesiologist grading criteria
BMI	Body Mass Index
CRP	C-reactive protein
ICD	International Classification of Diseases
ICU	Intensive Care Unit
IVIG	Intravenous immunoglobulin
NSTI	Necrotizing soft tissue infections
SIRS	Systemic inflammatory response syndrome
